# Association of Brachial Ankle Pulse Wave Velocity With New Onset Stroke in Hypertensive Patients Aged Less Than 65 With Normal Fasting Glucose Among Chinese Community-Based Population

**DOI:** 10.3389/fendo.2021.828286

**Published:** 2022-01-25

**Authors:** Cao Li, Zhuo Wang, Shuai Liu, Shanshan Guo, Yun Song, Lishun Liu, Ziyi Zhou, Binyan Wang, Meiqing Huang, Ruiqing Wang, Lijie Zhai, Yiming Gao, Xianhui Qin, Xiaobin Wang, Huaguang Zheng, Zhigang Zhao

**Affiliations:** ^1^ Department of Pharmacy, Beijing Tiantan Hospital, Capital Medical University, Beijing, China; ^2^ Key Laboratory of Precision Nutrition and Food Quality, Ministry of Education, Department of Nutrition and Health, College of Food Sciences and nutritional engineering, China Agricultural University, Beijing, China; ^3^ State Key Laboratory of Natural Medicines, Research Center of Biostatistics and Computational Pharmacy, China Pharmaceutical University, Nanjing, China; ^4^ Institute of Biomedicine, Anhui Medical University, Hefei, China; ^5^ Department of Scientific Research, Shenzhen Evergreen Medical Institute, Shenzhen, China; ^6^ Graduate School at Shenzhen, Tsinghua University, Shenzhen, China; ^7^ Health Management Center, Beijing Tiantan Hospital, Capital Medical University, Beijing, China; ^8^ National Clinical Research Center for Kidney Disease, State Key Laboratory for Organ Failure Research, Division of Nephrology, Nanfang Hospital, Southern Medical University, Guangzhou, China; ^9^ Department of Population, Family and Reproductive Health, Johns Hopkins University Bloomberg School of Public Health, Baltimore, MD, United States

**Keywords:** brachial ankle pulse wave velocity, new onset stroke, hypertensive patients, glucose, age

## Abstract

**Background:**

Previous studies have shown an association of stroke and brachial ankle pulse wave velocity (baPWV). However, due to limitations on total population size and small numbers of stroke cases, lack of power has prevented further detection among subgroups such as age and laboratory testing.

**Methods:**

A total of 19217 participants including 660 incident stroke patients were pooled in the present study. Participants were divided to 2 groups, aged less than 65 years [56.0 (50.0, 61.0)] and aged 65 years or more [70.0 (67.0, 74.0)].

**Results:**

After adjustment for demographic, anthropometric, and laboratory parameters, the incident stroke was positively associated to baPWV in the group aged less than 65 years (OR, 1.16; 95% CI, 1.05–1.28), but not in the older group aged 65 or more. When baPWV was assigned as quartiles, a significant, increased risk of new-onset stroke was found in quartiles 3-4 compared with quartile 1. In addition, the predictive value of baPWV for incident stroke was modified by fasting glucose in participants aged less than 65 years (*P*-interaction = 0.010). An increase in baPWV was strongly, positively associated to new-onset stroke in the subgroup of normal fasting glucose (< 5.6 mmol/L) (OR, 1.34; 95% CI, 1.15 - 1.57), but no effect was seen in the impaired fasting glucose (5.6-7.0 mmol/L) or diabetic fasting glucose (> 7.0 mmol/L) subgroups.

**Conclusions:**

Increased baPWV was significantly associated with new-onset stroke in a hypertensive population aged less than 65 years. Particularly, it is of great importance to monitor baPWV for predicting incident stroke in “relatively healthy” hypertensive patients, i.e. aged less than 65 years with normal fasting glucose.

## Introduction

Stroke ranks second among all causes of death, accounting for severe healthcare issues and huge financial burdens worldwide (1). The crude death rates of stroke have declined among patients over age 65, while the declines are modest among patients less than age 65; of note, from 2011 – 2016, the death rate flattened in patients between the ages of 45-54 and even increased in patients aged 55-64 (2). In addition, the incidence and prevalence of stroke have increased rapidly because of the ageing population in China, which accounts for approximately one-third of global stroke deaths (3). Thus, a predictive marker for determining those patients with a greater risk of stroke is of prominent clinical significance, for preventing new onset of stroke and enhancing patient welfare.

Arterial stiffness has been demonstrated to be a major risk factor for cardiovascular and neurological disorders such as diabetes, hypertension, atherosclerosis, cerebral small vessel disease, and stroke (4). Brachial ankle pulse wave velocity (baPWV) is a frequently used parameter for monitoring arterial stiffness due to its non-invasive measurement and reproducibility (5). Pulse wave velocity (PWV) largely increases with age, or in hypertension, even independent of blood pressure (6). Previous studies have demonstrated that PWV is positively associated with new onset stroke in hypertensive individuals (7, 8). However, due to study limitations of total population size and small numbers of stroke events, lack of power has prevented any further determination of differences among subgroups such as age or laboratory tests, i.e., glucose, cholesterol, and triglyceride levels, etc. Additionally, studies have focused on interactions with baPWV and the prognostic significance of stroke outcome (9–11), but researches of baPWV in predicting acute stroke are lacking.

In order to address the above-mentioned gap and illuminate new predictors of new-onset stroke, the present study aimed to (1) further investigate the role of baPWV in predicting stroke incidence, (2) evaluate whether risk predicting differs among sub-populations, and (3) find any potential effect modifiers on the predictive significance of baPWV, using data from a large-scale population study of 19217 hypertensive patients including 660 new-onset stroke patients.

## Methods

### Study Population and Design

The study population was pooled from the China H-type Hypertension Registry Study (CHHRS; URL: http://www.chictr.org.cn; Unique identifier: ChiCTR1800017274), which is an ongoing community-based non-intervention, prospective, observational, multicenter, real-world registry study and was mainly conducted in Rongcheng County, Shandong Province, and Lianyungang, Jiangsu Province, China. Eligible participants were over 18 years of age with hypertension, which was defined as seated, resting systolic blood pressure (SBP) ≥140 mmHg and/or diastolic blood pressure (DBP) ≥90 mmHg at both the screening and recruitment visit, or who were taking anti-hypertensive medications. Participants were excluded if they were unable to give informed consent or to participate in the follow-up according to the study protocol (**Supplementary Figure 1**). The investigators completed a standardized electronic medical record collection at baseline and at follow-up visits, which occurred every 3 months, for up to 3 years. At each visit, participants underwent a physical examination, and clinical outcomes were recorded. The present study was approved by the Ethics Committee of the Institute of Biomedicine, Anhui Medical University, Hefei, China. All participants signed an approved written consent form after the study protocol was thoroughly explained to them.

The primary outcome was an incident fatal or non-fatal symptomatic stroke, excluding subarachnoid hemorrhage or silent stroke (subclinical stroke); first ischemic stroke (fatal and nonfatal) and first hemorrhagic stroke (fatal and nonfatal) were secondary outcomes. Information on incident stroke for all participants was obtained from the Lianyungang CDC, and checked against a national health insurance system with electronic linkage to all hospitalizations, or was ascertained through active follow-up. In China, local public medical institutions are required to report all new onset stroke events to the local CDC. A report card including demographics, diagnosis, and date of stroke is required to be routinely submitted on the 28th of each month. Trained officials and the local CDC were responsible for quality control, such as characterizing and deleting repeated cases, error checking, and finding missed cases. Additionally, 5 percent of all cases were randomly selected for further validation by telephone or home-visit interviews (12).

### Laboratory Tests and Data Collection

Baseline characteristics were conducted by trained staff in accordance with standardized operating procedures. Participants were interviewed *via* a standard questionnaire for the current study, which included information on demographics, history of cigarette smoking, alcohol drinking, and medication usage. Anthropometric data, and physical and clinical characteristics were routinely measured by trained staff. Body mass index (BMI) was calculated as body weight in kilograms divided by the square of height in meters (kg/m^2^). Blood samples were collected from all participants after overnight fasting for clinical biomedical testing. Serum lipids, and fasting glucose were measured using automatic clinical analyzers (Beckman Coulter) at the Shenzhen Tailored Medical laboratory in Shenzhen, China.

### Measurements of baPWV

Level of baPWV was measured by a PWV/ABI instrument (form PWV/ABI, BP-203RPE; Omron-Colin, Japan) by trained technicians (7). After a minimum of 15 mins of rest, supine patients were fitted with oscillometric cuffs on ankles and bilateral brachia, with ECG electrodes on bilateral wrists. The semiconductor pressure sensor was used for recording pulse volume waveforms. Data of brachium and ankle volume waveforms were recorded in a 10s sampling interval with automatic adjustment of quality and gain. The time interval from brachium to ankle (ΔTba) was determined from the interval between the wave front of the brachial waveform and that of the ankle waveform. An automatic adjustment was applied to the distance between sampling points of baPWV by participant height. The path lengths from the suprasternal notch to the ankle (La) and from the suprasternal notch to the brachium (Lb) were determined by the following formula: La = 0.8129 × height (cm) + 12.328 and Lb = 0.2195 × height (cm) - 2.0734, respectively. Level of baPWV was calculated as: baPWV = (La−Lb)/Tba.

### Statistics

Data were presented as median ± interquartile range (IQR) for continuous variables and as frequency (%) for categorical variables. Population baseline characteristics of the different groups were analyzed using *t* tests, ANOVA tests, or χ2 tests, respectively. Baseline characteristics of participants were compared using 2-sample t tests, signed-rank tests, or χ2 tests between different groups, respectively. The relationship of baPWV and incident stroke was evaluated using multivariable logistic regression models and generalized linear regression models, with or without adjustment for age, sex, body mass index, systolic blood pressure, diastolic blood pressure, smoking status, alcohol consumption, baseline fasting blood glucose, total cholesterol, triglycerides, homocysteine in model 1, and additionally, history of antihypertensive drug use in model 2. For further exploratory analysis, interaction testing and stratified analyses were used to detect possible modifications on the association between baPWV and incident stroke. A 2-tailed *P* < 0.05 was considered to be statistically significant in the present study. Data were analyzed by statistical package R (http://www.r-project.org) and Empower (R) (www.empowerstats.com; X&Y Solutions, Inc., Boston, MA).

## Results

### Baseline Characteristics of the Study Participants

A total of 19217 participants including 660 new-onset stroke patients were registered in the present study. Characteristics of these participants stratified by age are illustrated in **Table 1**. Among those aged less than 65, compared with control participants [12061 patients aged 56.0 (50.0, 61.0)], new-onset stroke patients 320 subjects aged [59.0 (54.0, 62.0)] were older and had higher levels of SBP, DPB, baPWV, fasting glucose, triglycerides, and blood homocysteine. However, among participants aged 65 or more, no significant differences in SBP, DPB, baPWV, fasting glucose, and triglycerides between control participants [6496 subjects aged 70.0 (67.0, 74.0)] or stroke patients [340 subjects aged 71.0 (67.0, 76.0)] were found.

**Table 1:** Baseline characteristics of study participants stratified by age group^A^.

Characteristics	Age < 65 years			Age ≥ 65 years		
	Stroke cases	Non-stroke	*P*	Stroke cases	Non-stroke	*P*
Participants, *n*	320 (2.6)	12061		340 (5.0)	6496	<0.001^B^
Male	121 (37.8)	4115 (34.1)	0.188	148 (43.5)	2575 (39.6)	0.170
Age, y	59.0 (54.0, 62.0)	56.0 (50.0, 61.0)	<0.001	71.0 (67.0, 76.0)	70.0 (67.0, 74.0)	<0.001
BMI, kg/m^2^	26.1 (23.5, 28.4)	26.2 (23.9, 28.7)	0.220	24.9 (22.4, 27.7)	25.1 (22.7, 27.7)	0.348
SBP, mmHg	148.3 (139.3, 154.0)	145.3 (136.3, 152.0)	<0.001	147.3 (139.7,153.7)	146.7 (140.3, 152.7)	0.526
DBP, mmHg	97.0 (88.7, 105.7)	93.7 (86.3, 101.7)	<0.001	91.0 (82.3, 99.3)	89.7 (81.7, 97.7)	0.110
baPWV, cm/s	1701.1 (1523.2, 1921.8)	1588.0 (1403.0, 1759.0)	<0.001	1893.0 (1666.2, 2146.0)	1849.5 (1644.8, 2109.0)	0.048
Baseline laboratory results						
Fasting glucose, mmol/L	5.4 (4.9, 6.3)	5.3 (4.9, 5.9)	0.032	5.4 (4.9, 6.3)	5.4 (5.0, 6.1)	0.747
Total cholesterol, mmol/L	4.7 (4.2, 5.6)	4.7 (4.1, 5.4)	0.163	4.6 (4.1, 5.3)	4.8 (4.1, 5.5)	0.121
Triglycerides, mmol/L	1.7 (1.2, 2.5)	1.6 (1.1, 2.3)	0.046	1.4 (1.0, 2.1)	1.5 (1.0, 2.1)	0.534
Homocysteine, μmol/L	12.6 (10.2, 14.8)	12.0 (10.0,14.6)	0.075	14.5 (12.1, 17.7)	13.8 (11.4, 16.7)	0.005
Smoking status			0.147			0.649
Never	257 (80.3)	9822 (81.4)		246 (72.4)	4813 (74.1)	
Former	12 (3.8)	666 (5.5)		33 (9.7)	641 (9.9)	
Current	51 (15.9)	1573 (13.0)		61 (17.9)	1042 (16.0)	
Alcohol drinking status			0.623			0.661
Never	248 (77.5)	9217 (76.4)		253 (74.4)	4880 (75.1)	
Former	15 (4.7)	480 (4.0)		25 (7.4)	399 (6.1)	
Current	57 (17.8)	2364 (19.6)		62 (18.2)	1217 (18.7)	
Hypertensive	319 (99.7)	11704 (97.0)	0.009	330 (97.1)	6365 (98.0)	0.330
History of diseases						
Hypertension	275 (85.9)	9665 (80.1)	0.012	295 (86.8)	5399 (83.1)	0.092
Diabetes	31 (9.7)	807 (6.7)	0.046	31 (9.1)	544 (8.4)	0.703
Hyperlipidemia	39 (12.2)	1357 (11.3)	0.665	37 (10.9)	575 (8.9)	0.238
History of drug treatments						
Antihypertensive	187 (58.4)	5686 (47.1)	<0.001	204 (60.0)	3639 (56.0)	0.166

BMI indicates body mass index; SBP, systolic blood pressure; DBP, diastolic blood pressure; baPWV, brachial ankle pulse wave velocity. Data are presented as median (IQR)^A^ or n (%), unless otherwise indicated. ^B^Statistic differences was compared with rate of stroke cases in Age < 65 years or Age ≥ 65 years.

### baPWV Predicts New Onset Stroke in Hypertensive Subjects Aged Less Than 65 Years

**Figure 1:**
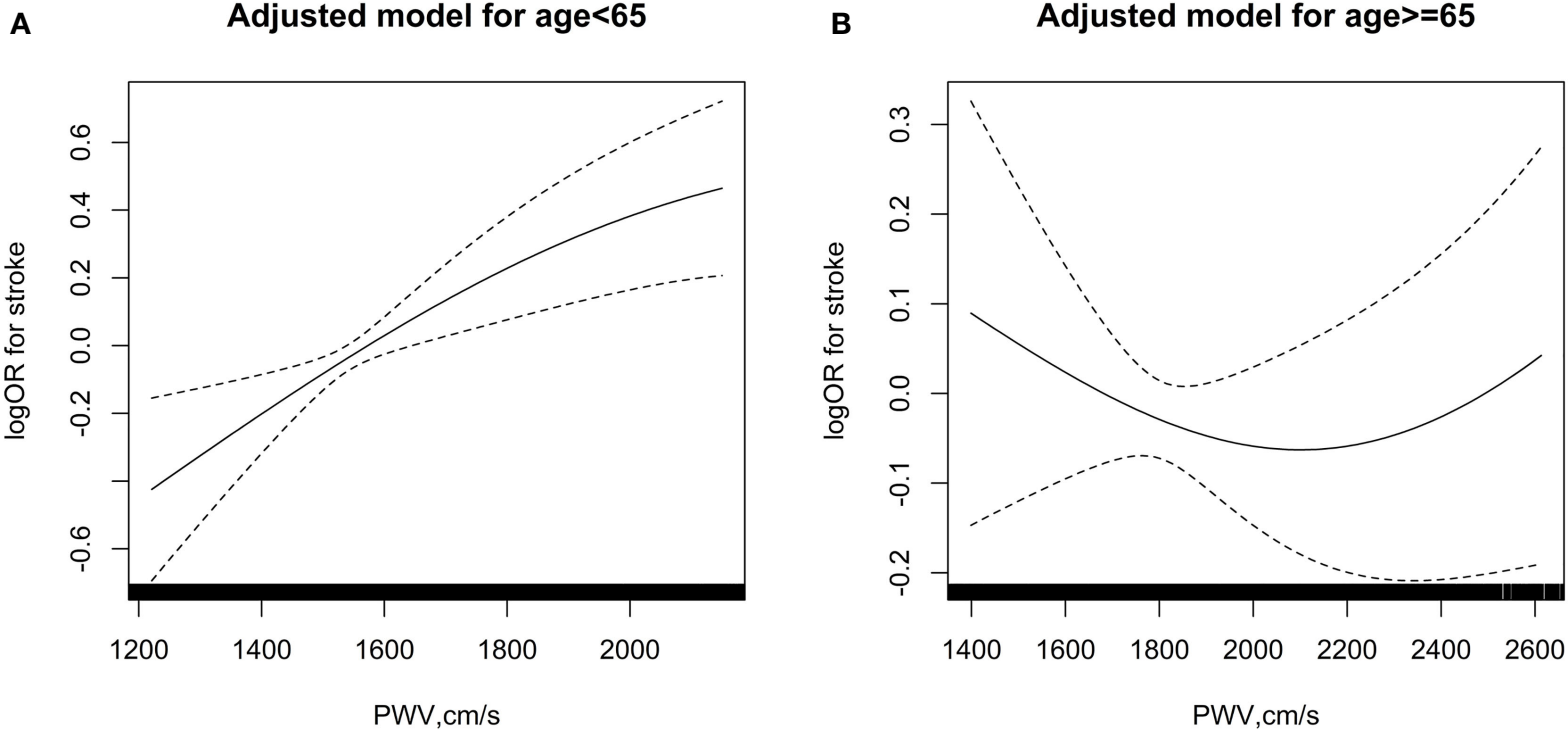
The association between baPWV and risk of first stroke in various age groups. **(A)** age < 65 years; **(B)** age≥ 65 years. The splines were adjusted for age, sex, body mass index, systolic blood pressure, diastolic blood pressure, smoking status and alcohol consumption, baseline fasting blood glucose, total cholesterol, triglycerides, homocysteine and history of antihypertensive drug use.

To determine the association of baPWV and age in predicting incident stroke, participants were separated into 2 groups: those aged less than 65 years, and those aged 65 years or more. Each group was further divided into quartiles (Q1-Q4) according to level of baPWV (**Table 2**). Cox regression was performed to investigate the effect of age and baPWV on new onset stroke. Overall, increased baPWV was positively associated to incident stroke (per SD: OR, 1.37; 95% CI, 1.26-1.50) for those aged less than 65 years in the crude model. A similar positive association of baPWV with stroke incidence in the younger group was observed in both model 1 (per SD: OR, 1.17; 95% CI, 1.06-1.29) and in model 2 (per SD: OR, 1.16; 95% CI, 1.05-1.28) (**Figure 1A**, **Table 2**). In contrast, an increase in baPWV was not associated with stroke incidence in patients aged 65 or more (**Figure 1B**, **Table 2**).

**Table 2:** The association between baseline brachial ankle pulse wave velocity (baPWV) and risk of first stroke in various age groups.

Age < 65 years								Age ≥ 65 years							
baPWV, cm/s	Cases (%)	Crude model		Adjusted model 1		Adjusted model 2		baPWV, cm/s	Cases (%)	Crude model		Adjusted model 1		Adjusted model 2	
		OR (95%CI)	*P*	OR (95%CI)	*P*	OR (95%CI)	*P*			OR (95%CI)	*P*	OR (95%CI)	*P*	OR (95%CI)	*P*
per SD increment	320 (2.6)	1.37 (1.26,1.50)	<0.001	1.17 (1.06,1.29)	0.002	1.16 (1.05,1.28)	0.003	per SD increment	340 (5.0)	1.13 (1.03,1.25)	0.011	1.06 (0.94,1.19)	0.354	1.05 (0.93,1.19)	0.393
Quartiles								Quartiles							
Q1 (<1406)	40 (1.3)	ref		ref		ref		Q1 (<1643)	80 (4.7)	ref		ref		ref	
Q2 (1406-1563)	49 (1.6)	1.23 (0.81,1.87)	0.339	0.97 (0.63,1.51)	0.900	0.96 (0.62,1.49)	0.857	Q2 (1643-1851)	68 (4.0)	0.84 (0.60,1.17)	0.303	0.68 (0.48,0.97)	0.033	0.68 (0.48,0.97)	0.032
Q3 (1563-1763)	105 (3.4)	2.68 (1.86,3.88)	<0.001	1.81 (1.21,2.70)	0.004	1.79 (1.20,2.66)	0.005	Q3 (1851-2110)	94 (5.5)	1.18 (0.87,1.60)	0.289	0.96 (0.69,1.34)	0.816	0.96 (0.69,1.33)	0.799
Q4 (≥1763)	126 (4.1)	3.23 (2.26,4.63)	<0.001	1.85 (1.21,2.82)	0.004	1.81 (1.19,2.77)	0.006	Q4 (≥2110)	98 (5.7)	1.23 (0.91,1.67)	0.174	0.93 (0.65,1.33)	0.693	0.93 (0.65,1.32)	0.681
*p* for trend			<0.001		<0.001		<0.001	*p* for trend			0.050		0.809		0.825

Model 1 is adjusted for age, sex, body mass index, systolic blood pressure, diastolic blood pressure, smoking status, alcohol consumption, baseline fasting blood glucose, total cholesterol, triglycerides, and homocysteine.

Model 2 is adjusted for age, sex, body mass index, systolic blood pressure, diastolic blood pressure, smoking status, alcohol consumption, baseline fasting blood glucose, total cholesterol, triglycerides, homocysteine, and history of antihypertensive drug use.

In participants aged less than 65 years, compared with quartile 1 (Q1), the odds ratios of incident stroke increased along with increased baPWV in the crude model (Q3: OR, 2.68, 95% CI, 1.86 - 3.88, P < 0.001; Q4: OR, 3.23, 95% CI, 2.26 - 4.63, P < 0.001) and in the adjusted model 1 and model 2 (**Table 2**). However, these associations of baPWV with stroke were absent in patients aged 65 or more.

### Fasting Glucose Modifies the Association Between New-Onset Stroke and Elevated Arterial Stiffness in Subjects Aged Less Than 65 Years

To analyze potential modifiable risk factors in the association between incident stroke and baPWV, a stratified sub-analysis was further performed in participants stratified by age group (aged less than 65 or aged 65 or more), and the subgroups sex, body mass index, systolic blood pressure, diastolic blood pressure, smoking status, alcohol consumption, baseline fasting blood glucose, total cholesterol, triglycerides, homocysteine, and history of antihypertensive use (**Table 3**). Notably, only fasting glucose had a significant, modifiable effect on the association between new-onset stroke and baPWV in the younger age group (*P* for interaction, 0.010), but not in the older group (*P* for interaction, 0.233).

**Table 3:** The association between baPWV and risk of first stroke in various subgroups stratified by age.

Subgroups	Age < 65 years			Age ≥ 65 years		
	Cases (%)	OR (95% CI)	*P* for interaction	Cases (%)	OR (95% CI)	*P* for interaction
Sex			0.917			0.780
Male	121 (2.9)	1.16 (0.98,1.38)		148 (5.4)	1.00 (0.83,1.20)	
Female	199 (2.4)	1.23 (1.03,1.48)		192 (4.7)	1.08 (0.93,1.24)	
Body mass index, kg/m^2^			0.566			0.527
<24	96 (3.0)	1.21 (0.94,1.55)		136 (5.3)	0.99 (0.83,1.18)	
≥24	221 (2.4)	1.21 (1.05,1.39)		199 (4.8)	1.10 (0.95,1.26)	
SBP, mmHg			0.491			0.735
<140	88 (2.2)	1.25 (0.89,1.76)		86 (5.3)	1.01 (0.77,1.32)	
≥140	231 (2.7)	1.20 (1.06,1.37)		253 (4.9)	1.06 (0.94,1.19)	
DBP, mmHg			0.152			0.376
<90	88 (2.0)	1.50 (1.09,2.08)		155 (4.6)	0.94 (0.77,1.16)	
≥90	232 (2.9)	1.20 (1.05,1.36)		184 (5.4)	1.13 (0.99,1.27)	
Smoking status			0.989			0.125
Never	257 (2.5)	1.22 (1.04,1.44)		246 (4.9)	1.06 (0.94,1.20)	
Former	12 (1.8)	1.37 (0.71,2.66)		33 (4.9)	0.70 (0.43,1.15)	
Current	51 (3.1)	1.16 (0.94,1.45)		61 (5.5)	1.18 (0.89,1.56)	
Alcohol drinking status			0.191			0.290
Never	248 (2.6)	1.20 (1.02,1.42)		253 (4.9)	0.98 (0.85,1.11)	
Former	15 (3.0)	2.20 (1.13,4.35)		25 (5.9)	1.54 (1.03,2.33)	
Current	57 (2.4)	1.10 (0.88,1.39)		62 (4.8)	1.25 (0.96,1.63)	
Fasting glucose, mmol/L			0.010			0.233
<5.6	188 (2.4)	1.34 (1.15,1.57)		188 (4.8)	0.92 (0.77,1.09)	
5.6 to <7.0	74 (2.3)	1.14 (0.83,1.56)		99 (5.1)	1.11 (0.90,1.36)	
≥7.0 or diabetes	56 (4.2)	0.93 (0.66,1.31)		48 (5.2)	1.19 (0.96,1.36)	
Total cholesterol, mmol/L			0.502			0.589
<5.2	209 (2.5)	1.26 (1.10,1.46)		232 (5.2)	1.09 (0.95,1.25)	
≥5.2	109 (2.8)	1.05 (0.83,1.33)		101 (4.4)	0.94 (0.77,1.15)	
Triglycerides, mmol/L			0.408			0.320
<1.7	155 (2.4)	1.16 (0.97,1.39)		204 (5.1)	1.01 (0.87,1.17)	
≥1.7	155 (2.8)	1.25 (1.05,1.50)		123 (4.7)	1.09 (0.93,1.28)	
Homocysteine, *μ*mol/L			0.627			0.415
<15	235 (2.5)	1.19 (1.04,1.37)		181 (4.4)	1.03 (0.87,1.19)	
≥15	79 (2.9)	1.24 (0.96,1.60)		153 (5.9)	1.06 (0.90,1.25)	

Each subgroup analysis was adjusted, if not stratified for age, sex, body mass index, systolic blood pressure (SBP), diastolic blood pressure (DBP), smoking status, alcohol consumption, baseline fasting blood glucose, total cholesterol, triglycerides, homocysteine, and history of antihypertensive drug use. Data are presented as median (IQR) or n (%), unless otherwise indicated.

Particularly, the increase of baPWV was strongly, positively associated to new-onset stroke in the subgroup with normal fasting glucose (fasting glucose < 5.6 mmol/L) for those aged less than 65 (OR, 1.34; 95% CI, 1.15–1.57), but the effect was not seen in those with either impaired fasting glucose (fasting glucose: 5.6-7.0 mmol/L) (OR, 1.14; 95% CI, 0.83-1.56) or diabetic fasting glucose (fasting glucose > 7.0 mmol/L) (OR, 0.93; 95% CI, 0.66-1.31) (**Table 3**). In addition, for those aged less than 65, the level of baPWV was significantly higher in stroke patients [baPWV: 1676.5 (1509.8, 1924.2) cm/s] compared with non-stroke control patients [baPWV: 1528.7 (1377.0, 1715.0)] (*P* < 0.001) in the normal glucose group; the level of baPWV was also higher in stroke patients [baPWV: 1724.0 (1580.2, 1854.5) cm/s] compared with controls [baPWV: 1588.5 (1432.0, 1797.0) cm/s] (*P* < 0.001) in the impaired fasting glucose group, while there was no statistical difference between cases and controls among diabetic fasting glucose patients (*P* = 0.431) (**Supplementary Table 1**).

## Discussion

The present study revealed two new observations (1) new-onset stroke was positively associated to increased arterial stiffness in hypertensive patients aged less than 65, but not in those aged 65 or more, and (2) the predicting role of baPWV on incident stroke was modified by fasting glucose in younger patients (aged less than 65), but not in older patients; notably, a significant predictive value of baPWV for incident stroke was found in hypertensive patients with normal fasting glucose.

To the best of our knowledge, this cohort included the largest number of incident stroke cases for the assessment of the association between stroke and arterial stiffness. Both pioneer and recent studies have confirmed that arterial stiffness measured by carotid-femoral pulse wave velocity (cfPWV) independently predicts cardiovascular diseases, including first stroke in hypertensive patients (8, 13, 14). However, our current study indicates that the predictive value of arterial stiffness was modified by glucose in patients aged less than 65 years. There could be multiple potential mechanisms to explain these findings. First, although both cfPWV and baPWV are used for measurement of central artery stiffness and are significantly positively associated for predicting cardiovascular diseases (CVDs), baPWV is considered moderately associated to peripheral artery stiffness (15); in comparison to cfPWV, baPWV has shown a greater association with left ventricle mass, cardiovascular function, and coronary calcium (16, 17). The second possible reason might be due to characteristics of the study population. In our study, we divided study participants into two age groups: those under age 65 and those aged 65 or more, with age at 56.0 and 70.0 years, respectively (**Supplementary Table 2**); while patients from previous studies had age of 50 ± 13 (13), 51.05 ± 12.64 (8), and 51 ± 13 years (14). The base-line characteristics of stroke cases was shown in **Supplementary Table 3**. Interestingly, these results suggest a better prediction of PWV for first-ever stroke in younger compared with older individuals, which is consistent with recent findings from a meta-analysis of 17, 635 subjects (18).

Intriguingly, our current study clearly indicates a predictive value of baPWV in “relatively healthy” hypertensive patients, i.e., aged less than 65 years with normal fasting glucose. Arterial stiffness, a predictor of all-cause mortality and CVD events (18, 19), is positively related to increased age. Particularly, PWV levels significantly increase more steeply in hypertensive patients aged more than 50 years old compared to patients aged 50 or less (20, 21). As a result of a substantial increase of arterial stiffness in older patients aged 65 or more, we presumed that the predictive value of PWV among stroke and non-stroke patients might attenuate or even disappear. Multiple mechanisms could explain the association of PWV with first stroke. First, increased arterial stiffness relates to decreased regional cerebral blood flow and higher cerebrovascular reactivity (22), and these alterations of cerebrovascular hemodynamics and arteriole damage may contribute to injury of the central nervous system and, consequently, stroke. As level of PWV accumulates overtime, endothelial function is impaired in patients with acute stroke (23). Second, increased arterial stiffness also reflects stenosis of the peripheral arteries, which favors the likelihood of cardiovascular diseases (24).

Fasting blood glucose was a modifiable factor for the predictive value of baPWV in patients aged less than 65 years in our present study (**Table 3**). Increased arterial stiffness, measured by baPWV or cfPWV, has been shown to be positively associated with the risk of incident diabetes (25, 26); moreover, our findings suggest that arterial stiffness, in combination with fasting glucose, plays a role in predicting stroke: in hypertensive patients aged less than 65 years, level of baPWV was significantly higher in incident stroke patients compared to non-stroke controls with normal fasting glucose or impaired fasting glucose, while these effects were absent in older patients (**Supplementary Table 1**). In a large-scale study including 698782 participants, fasting glucose levels in individuals without diabetes had no significant improvement for predicting vascular disorders when status of conventional risk factors was provided (27). Our results from persons without diabetes provide a possibility to detect incident stroke, although the precise mechanism needs to be further determined.

Many studies indicate that the rise of incident stroke in young and middle-aged adults has become a critical problem in western countries and China (reviewed in (3, 28)). Of significance, the average age of Chinese stroke cases is about 10 years younger than that of western countries (29). Approximately one third of stroke patients are under age 60 in China, bringing significant losses to people of working age and their families (30). Our findings may provide evidence for improving upon the prevention of incident stroke in hypertensive middle-aged adults. In addition, the INTERSTROKE project suggests that hypertension still ranks as the number 1 substantial risk factor for acute stroke (31). In patients with hypertension, those with appropriate control of hypertension have lower baPWV levels and a decreased risk of first stroke.

Taken together, our study suggests that arterial stiffness is positively associated with new-onset stroke in hypertensive patients aged less than 65 years. Additionally, fasting glucose level is a modifiable factor of baPWV in predicting stroke, i.e., an independent predictive significance was found in middle-aged hypertensive patients with normal fasting glucose. Our findings may provide evidence of clinical prevention and a potential therapeutic target for incident stroke. Targeted monitoring and intervention for decreasing baPWV might be an efficient strategy in the fight against incident stroke, to reduce a possible disability-adjusted life year in “relatively healthy” hypertensive patients, and ultimately, to achieve higher social and economic gains.

The present study has some limitations. First, the follow-up time was not long (3 years); further long-term studies should be performed. Second, the participants were from China which limits the generalizability of the results to other populations. Third, patients pooled in this study were taking different types of antihypertensive medications; since these medications might have different effects on arterial stiffness, further sub-group studies are required.

## Data Availability Statement

The original contributions presented in the study are included in the article/**Supplementary Material**. Further inquiries can be directed to the corresponding authors.

## Ethics Statement 

The studies involving human participants were reviewed and approved by Anhui Medical University, Hefei, China. The patients/participants provided their written informed consent to participate in this study.

## Author Contributions

CL, ZhZ, HZ, and XW conceived and designed the study. CL wrote the manuscript. ZW, SL, YS, LL, ZiZ, MH, and LL performed statistics and generated the figure. All authors listed have made a substantial, direct and intellectual contribution to the work, and approved it for publication.

## Funding

The study was supported by MiaoPu Project of Beijing Tiantan Hospital (2020MP07); Yangfan Project of Beijing Hospitals Authority (ZYLX201827); Beijing Medical and Health Foundation (YWJKJJHKYJJ-B182875); China National Key Research and Development Program (2018YFC1311203), the National Key Research and Development Program (2016YFE0205400, 2018ZX09739010, 2018ZX09301034003); the Department of Science and Technology of Guangdong Province (2020B121202010); the Science and Technology Planning Project of Guangzhou, China (201707020010); the Science, Technology and Innovation Committee of Shenzhen (GJHS20170314114526143, JSGG20180703155802047); the Economic, Trade and Information Commission of Shenzhen Municipality (20170505161556110, 20170505160926390, 201705051617070).

## Conflict of Interest

The authors declare that the research was conducted in the absence of any commercial or financial relationships that could be construed as a potential conflict of interest.

## Publisher’s Note

All claims expressed in this article are solely those of the authors and do not necessarily represent those of their affiliated organizations, or those of the publisher, the editors and the reviewers. Any product that may be evaluated in this article, or claim that may be made by its manufacturer, is not guaranteed or endorsed by the publisher.
