# Supplementary material for: Association of Brachial Ankle Pulse Wave Velocity With New Onset Stroke in Hypertensive Patients Aged Less Than 65 With Normal Fasting Glucose Among Chinese Community-Based Population
Source: Front Endocrinol (Lausanne). 2022 Jan 25;12:828286. doi: 10.3389/fendo.2021.828286 (PMC8822600; doi:10.3389/fendo.2021.828286)
Supplement: Supplementary file 1 [file DataSheet_1.docx]

**Supplementary Figure 1.** Flow chart of the participants.

Participants excluded for missing laboratory, ID, or PWV data

PWV screening for Lianyungang cohort in 2016 (N=23283)

Stroke cases from Lianyungang CDC in 2013-2018 (N=24303)

Lianyungang PWV Cohort (N=19217)

New Onset Cases (N=660)

Non-Stroke (N=18557)

**Supplementary Table 1**

Baseline characteristics of study participants stratified by fasting glucose concentrations in the less than 65 years age group

| Age < 65 years | | | | | | | | | |
| --- | --- | --- | --- | --- | --- | --- | --- | --- | --- |
| Characteristics | GLU < 5.6 mmol/L | | | 5.5 ≤ GLU < 7.0 mmol/L | |  | GLU ≥ 7.0 mmol/L or Diabetes | | |
|  | Stroke Cases | Non-stroke | *P* | Stroke Cases | Non-stroke | *P* | Stroke Cases | Non-stroke | *P* |
| Participants, *n* | 188 | 7506 |  | 74 | 3122 |  | 56 | 1287 |  |
| Male, *n (%)* | 67 (35.6) | 2392(31.9) | 0.310 | 30 (40.5) | 1196 (40.5) | 0.788 | 23 (41.1) | 464 (36.1) | 0.533 |
| Age, y | 59.0 (54.0, 62.0) | 54.0 (50.0, 60.0) | <0.001 | 60.0 (54.0, 63.0) | 57.0 (51.0, 61.0) | <0.001 | 58.5 (53.8, 62.0) | 58.0 (52.0, 62.0) | 0.295 |
| BMI, kg/m^2^ | 25.5 (22.9, 28.2) | 26.0 (23.7, 28.5) | 0.039 | 26.1 (24.2, 27.9) | 26.5 (24.2, 28.9) | 0.453 | 27.8 (25.6, 29.5) | 26.9 (24.5, 29.5) | 0.285 |
| SBP, mmHg | 148.3 (139.3, 153.8) | 144.3 (135.0, 151.0) | <0.001 | 148.7 (142.7, 154.3) | 146.3 (138.3, 153.0) | 0.044 | 145.7 (136.8, 153.2) | 147.0 (139.3, 153.9) | 0.487 |
| DBP, mmHg | 97.5 (89.7, 106.0) | 93.7 (86.3, 101.3) | <0.001 | 99.2 (89.0, 106.9) | 94.0 (87.0, 102.3) | 0.008 | 93.0 (86.8, 102.8) | 93.7 (85.7, 102.0) | 0.831 |
| baPWV, cm/s | 1676.5 (1509.8, 1924.2) | 1528.0 (1377.0, 1715.0) | <0.001 | 1724.0 (1580.2, 1854.5) | 1588.5 (1432.0, 1797.0) | 0.001 | 1672.0 (1566.2, 1977.8) | 1681.0 (1510.0, 1909.0) | 0.431 |
| Baseline laboratory results | |  |  |  |  |  |  |  |  |
| Fasting glucose, mmol/L | 5.0 (4.7, 5.3) | 5.0 (4.7, 5.3) | 0.855 | 6.1 (5.7, 6.5) | 6.0 (5.8, 6.3) | 0.278 | 9.6 (7.8, 11.4) | 8.5 (7.6, 10.7) | 0.097 |
| Total cholesterol, mmol/L | 4.7 (4.2, 5.6) | 4.6 (4.0, 5.3) | 0.054 | 4.6 (4.1, 5.3) | 4.8 (4.2, 5.6) | 0.173 | 5.1 (4.2, 6.1) | 5.0 (4.3, 5.8) | 0.497 |
| Triglycerides, mmol/L | 1.5 (1.1, 2.4) | 1.5 (1.0, 2.2) | 0.254 | 1.7 (1.1, 2.5) | 1.6 (1.1, 2.4) | 0.988 | 2.1 (1.5, 3.1) | 2.0 (1.4, 3.1) | 0.226 |
| Homocysteine, μmol/L | 12.9 (10.5, 6.0) | 12.1 (10.1, 4.7) | 0.020 | 12.6 (10.1, 3.9) | 12.0 (9.9, 14.7) | 0.759 | 12.1 (9.4, 14.4) | 11.7 (9.7, 14.0) | 0.956 |
| Smoking status, *n (%)* |  |  | 0.471 |  |  | 0.796 |  |  | 0.028 |
| Never | 156 (83.0) | 6156 (82.1) |  | 57 (77.0) | 2503 (80.2) |  | 42 (75.0) | 1046 (81.3) |  |
| Former | 6 (3.2) | 383 (5.1) |  | 5 (6.8) | 188 (6.0) |  | 1 (1.8) | 83 (6.4) |  |
| Current | 26 (13.8) | 958 (12.8) |  | 12 (16.2) | 431 (13.8) |  | 13 (23.2) | 158(12.3) |  |
| Alcohol drinking status, *n (%)* |  |  | 0.726 |  |  | 0.275 |  |  | 0.489 |
| Never | 148 (78.7) | 5898 (78.6) |  | 56 (75.7) | 2248 (72.0) |  | 42 (75.0) | 977 (75.9) |  |
| Former | 9 (4.8) | 285 (3.8) |  | 5 (6.8) | 128 (4.1) |  | 1 (1.8) | 61 (4.7) |  |
| Current | 31 (16.5) | 1325 (17.7) |  | 13 (17.6) | 746 (23.9) |  | 13 (23.2) | 249 (19.3) |  |
| Hypertensive *n (%)* | 187 (99.5) | 7227 (96.3) | 0.035 | 74 (100.0) | 3073 (98.4) | 0.544 | 56 (100.0) | 1264 (98.2) | 0.629 |
| History of diseases |  |  |  |  |  |  |  |  |  |
| Hypertension | 156 (83.0) | 5947 (79.2) | 0.245 | 69 (93.2) | 2530 (81.0) | 0.012 | 49 (87.5) | 1075 (83.5) | 0.547 |
| Diabetes | 3 (1.6) | 139 (1.9) | 1.000 | 6 (8.1) | 177 (5.7) | 0.523 | 22 (39.3) | 491 (38.2) | 0.976 |
| Hyperlipidemia | 17 (9.0) | 719 (9.6) | 0.903 | 9 (12.2) | 367 (11.8) | 1.000 | 13 (22.2) | 257 (20.0) | 0.672 |
| History of drug treatments treatments |  |  |  |  |  |  |  |  |  |
| Antihypertensive | 106 (56.4) | 3398 (45.3) | 0.003 | 48 (64.9) | 1535 (49.2) | 0.011 | 33 (58.9) | 681 (52.9) | 0.456 |

BMI indicates body mass index; SBP, systolic blood pressure; DBP, diastolic blood pressure; baPWV, brachial ankle pulse wave velocity; Data are presented as median (IQR) or n (%), unless otherwise indicated.

**Supplementary Table 2**

Baseline characteristics of study participants stratified by age group^1^

| Characteristics | Overall | Age<65 years | Age>65 years | *P* |
| --- | --- | --- | --- | --- |
| Participants, *n* | 19217 | 12381 | 6836 |  |
| Male, *n (%)* | 6959 (36.2) | 4236 (34.2) | 2723 (39.8) | <0.001 |
| Age, y | 61.0 (54.0, 67.0) | 56.0 (50.0, 61.0) | 70.0 (67.0, 74.0) | <0.001 |
| BMI, kg/m^2^ | 25.8 (23.5, 28.4) | 26.2 (23.9, 28.7) | 25.1 (22.7, 27.7) | <0.001 |
| SBP, mmHg | 146.0 (137.7, 152.3) | 145.3 (136.7, 152.0) | 146.7 (140.3, 152.7) | <0.001 |
| DBP, mmHg | 92.7 (84.7, 100.7) | 93.7 (86.3, 101.7) | 90.0 (81.7, 98.0) | <0.001 |
| baPWV, cm/s | 1657.0 (1462.0, 1902.0) | 1563.0 (1406.0, 1763.0) | 1852.0 (1645.0,2111.0) | <0.001 |
| Baseline laboratory results |  |  |  |  |
| Fasting glucose, mmol/L | 5.4 (4.9, 6.0) | 5.3 (4.9, 5.9) | 5.4 (5.0, 6.1) | <0.001 |
| Total cholesterol, mmol/L | 4.7 (4.1, 5.4) | 4.7 (4.1, 5.4) | 4.8 (4.1, 5.5) | 0.002 |
| Triglycerides, mmol/L | 1.5 (1.1, 2.2) | 1.6 (1.1, 2.3) | 1.5 (1.0, 2.1) | <0.001 |
| Homocysteine, μmol/L | 12.7 (10.4, 15.5) | 12.1 (10.0, 14.6) | 13.8 (11.4, 16.8) | <0.001 |
| Smoking status |  |  |  | <0.001 |
| Never | 15138 (78.8) | 10079 (81.4) | 5059 (74.0) |  |
| Former | 1352 (7.0) | 678 (5.5) | 674 (9.9) |  |
| Current | 2727 (14.2) | 1624 (13.1) | 1103 (16.1) |  |
| Alcohol drinking status |  |  |  | <0.001 |
| Never | 14598 (76.0) | 9465 (76.4) | 5133 (75.1) |  |
| Former | 919 (4.8) | 495 (4.0) | 424 (6.2) |  |
| Current | 3700 (19.3) | 2421 (19.6) | 1279 (18.7) |  |
| Hypertensive | 18718 (97.4) | 12023 (97.1) | 6695 (97.9) | 0.001 |
| History of diseases |  |  |  |  |
| Hypertension | 15634 (81.4) | 9940 (80.3) | 5694 (83.3) | <0.001 |
| Diabetes | 1413 (7.4) | 838 (6.8) | 575 (8.4) | <0.001 |
| Hyperlipidemia | 2008 (10.4) | 1396 (11.3) | 612 (9.0) | <0.001 |
| History of drug treatments |  |  |  |  |
| Antihypertensive | 9716 (50.6) | 5853(47.4) | 3843 (56.2) | <0.001 |
| Incident stroke cases | 660 (3.4) | 320 (2.6) | 340 5.0) | <0.001 |

BMI indicates body mass index; SBP, systolic blood pressure; DBP, diastolic blood pressure; baPWV, brachial ankle pulse wave velocity; Data are presented as median (IQR)^1^ or n (%), unless otherwise indicated.

**Supplementary Table 3**

Baseline characteristics of stroke cases stratified by age group^1^

| Characteristics | Overall | Age<65 years | Age>65 years | *P* |
| --- | --- | --- | --- | --- |
| Participants, *n* | 660 | 320 | 340 |  |
| Male, *n (%)* | 269 (40.8) | 121 (37.8) | 148 (43.5) | 0.157 |
| Age, y | 65.0 (60.0, 72.0) | 59.0 (54.0, 62.0) | 71.0 (67.0, 76.0) | <0.001 |
| BMI, kg/m^2^ | 25.6 (3.8) | 26.1 (3.5) | 25.1 (3.9) | 0.001 |
| SBP, mmHg | 147.7 (139.4, 153.7) | 148.3 (139.3, 154.0) | 147.3 (139.7, 153.7) | 0.659 |
| DBP, mmHg | 93.7 (85.2, 103.0) | 97.0 (88.7, 105.7) | 91.0 (82.3, 99.3) | <0.001 |
| baPWV, cm/s | 1786.5 (1589.5, 2043.5) | 1701.0 (1523.2, 1921.8) | 1893.0 (1666.2, 2146.0) | <0.001 |
| Baseline laboratory results |  |  |  |  |
| Fasting glucose, mmol/L | 5.4 (4.9, 6.3) | 5.4 (4.9, 6.3) | 5.4 (4.9, 6.3) | 0.858 |
| Total cholesterol, mmol/L | 4.7 (4.1, 5.5) | 4.7 (4.2, 5.6) | 4.6 (4.1, 5.3) | 0.149 |
| Triglycerides, mmol/L | 1.5 (1.1, 2.3) | 1.7 (1.2, 2.5) | 1.4 (1.0, 2.1) | <0.001 |
| Homocysteine, μmol/L | 13.3 (11.1, 16.6) | 12.6 (10.2, 14.8) | 14.5 (12.1, 17.7) | <0.001 |
| Smoking status |  |  |  | 0.006 |
| Never | 503 (76.2) | 257 (80.3) | 246 (72.4) |  |
| Former | 45 (6.8) | 12 (3.8) | 33 (9.7) |  |
| Current | 112 (17.0) | 51 (15.9) | 61 (17.9) |  |
| Alcohol drinking status |  |  |  | 0.34 |
| Never | 501 (75.9) | 248 (77.5) | 253 (74.4) |  |
| Former | 40 (6.1) | 15 (4.7) | 25 (7.4) |  |
| Current | 119 (18.0) | 57 (17.8) | 62 (18.2) |  |
| Hypertensive | 649 (98.3) | 319 (99.7) | 330 (97.1) | 0.02 |
| History of diseases |  |  |  |  |
| Hypertension | 570 (86.4) | 275 (85.9) | 295 (86.8) | 0.845 |
| Diabetes | 62 (9.4) | 31 (9.7) | 31 (9.1) | 0.907 |
| Hyperlipidemia | 76 (11.5) | 39 (12.2) | 37 (10.9) | 0.687 |
| History of drug treatments |  |  |  |  |
| Antihypertensive | 391 (59.2) | 187 (58.4) | 204 (60.0) | 0.742 |

BMI indicates body mass index; SBP, systolic blood pressure; DBP, diastolic blood pressure; baPWV, brachial ankle pulse wave velocity; Data are presented as median (IQR)^1^ or n (%), unless otherwise indicated.
